# Biophysical Approach to Mechanisms of Cancer Prevention and Treatment with Green Tea Catechins

**DOI:** 10.3390/molecules21111566

**Published:** 2016-11-18

**Authors:** Masami Suganuma, Atsushi Takahashi, Tatsuro Watanabe, Keisuke Iida, Takahisa Matsuzaki, Hiroshi Y. Yoshikawa, Hirota Fujiki

**Affiliations:** 1 Graduate School of Science and Engineering, Saitama University, Shimo-okubo 255, Sakura-ku, Saitama 338-8570, Japan; keyiida@mail.saitama-u.ac.jp; 2 Research Institute for Clinical Oncology, Saitama Cancer Center, Ina, Kitaadachi-gun, Saitama 362-0806, Japan; 3 Saitama Prefectural Tea Research Institute, Kamiyaganuki 244-2, Iruma, Saitama 358-0042, Japan; takahashi.atsushi@pref.saitama.lg.jp; 4 Faculty of Medicine, Saga University, Nabeshima, Saga 849-8501, Japan; TATSURO.WATANABE@ucdenver.edu (T.W.); uv4h-fjk@asahi-net.or.jp (H.F.); 5 Department of Chemistry, Saitama University, Shimo-okubo 255, Sakura-ku, Saitama 338-8570, Japan; taka.chem@gmail.com (T.M.); hiroshi@mail.saitama-u.ac.jp (H.Y.Y.)

**Keywords:** AFM, adhesion, durotaxis, EGCG, fluidity, membrane stiffness, metastasis, migration, rigidity, wound healing

## Abstract

Green tea catechin and green tea extract are now recognized as non-toxic cancer preventives for humans. We first review our brief historical development of green tea cancer prevention. Based on exciting evidence that green tea catechin, (−)-epigallocatechin gallate (EGCG) in drinking water inhibited lung metastasis of B16 melanoma cells, we and other researchers have studied the inhibitory mechanisms of metastasis with green tea catechins using biomechanical tools, atomic force microscopy (AFM) and microfluidic optical stretcher. Specifically, determination of biophysical properties of cancer cells, low cell stiffness, and high deformability in relation to migration, along with biophysical effects, were studied by treatment with green tea catechins. The study with AFM revealed that low average values of Young’s moduli, indicating low cell stiffness, are closely associated with strong potential of cell migration and metastasis for various cancer cells. It is important to note that treatments with EGCG and green tea extract elevated the average values of Young’s moduli resulting in increased stiffness (large elasticity) of melanomas and various cancer cells. We discuss here the biophysical basis of multifunctions of green tea catechins and green tea extract leading to beneficial effects for cancer prevention and treatment.

## 1. Introduction

**Figure 1:**
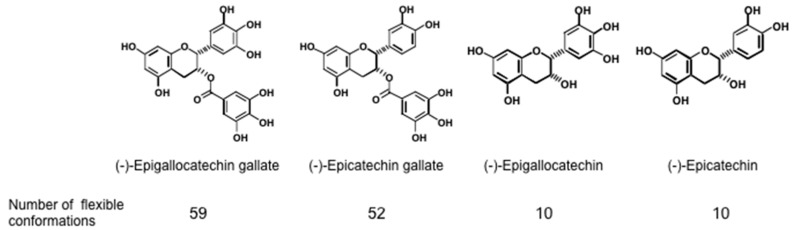
Structures of four green tea catechins and number of their flexible conformations.

Since green tea contains various cancer preventive catechins,—(−)-epigallocatechin gallate (EGCG), (−)-epigallocatechin (EGC), and (−)-epicatechin gallate (ECG) (Figure 1)—we theorised that drinking over 10 cups (120 mL/cup) of green tea per day could prevent cancer recurrence in various organs in humans. The phase II clinical prevention trials of colorectal adenoma recurrence in Japanese and Korean patients, along with prostate cancer patients with high-grade prostate intraepithelial neoplasia in Italy, showed the beneficial efficacy of green tea [1,2,3,4]. In 1987, we first reported the cancer preventive activity of EGCG [5], and since then, the significance of EGCG and green tea in cancer prevention and treatment has become widely recognized in the world [6,7]. We chronologically introduce here the historical development of green tea and EGCG as cancer preventives in humans.

This paper deals with our biophysical study of green tea catechins to elucidate which mechanisms can inhibit cancer metastasis. Recent advances in physical biomechanical tools, such as atomic force microscopy (AFM), have made it possible to quantitatively measure the biophysical properties of a single living cancer cell with high accuracy [8,9]. Study of cancer cells with AFM revealed that cancer cells are significantly softer than normal counterpart cells, and that less stiffness of cancer cells (softened cells) is associated with high migration potential (metastatic potential) of various cancer types. It is essential to note that treatment of cancer cells with EGCG increased cell stiffness (producing larger elasticity), and stiffened both cytosol and membrane, resulting in increased difficulty of cancer cells penetrating into adjacent tissues. This biophysical property is closely related to the proposed mechanism “sealing effects of EGCG” which we demonstrated in our experiments in 1992 [10].

We divide this review into six main sections: (1) Introduction; (2) Historical development; (3) Inhibition of metastasis with EGCG and green tea catechins; (4) Sealing effects of EGCG; (5) Significance of biophysical phenotypes in cancer progression and metastasis; and (6) Biophysical effects with green tea catechins.

## 2. Historical Development

Looking back at the beginning of this research, we first screened 30 polyphenols derived from medicinal plants in 1983, which were kindly provided by Takuo Okuda of the Faculty of Pharmaceutical Sciences, Okayama University. Our first assay was to test whether a compound could bind to the receptor of the tumor promoter, 12-*O*-tetradecanoylphorbol-13-acetate (TPA), in a particular fraction of mouse skin, and next to see whether the compound would inhibit the tumor promotion of TPA on mouse skin [5]. Although EGCG is structurally quite different from TPA, EGCG bound to the phorbol ester receptor about 340 times weaker than TPA did, and inhibited protein kinase C (PKC) activation induced by a TPA-type tumor promoter, teleocidin. This result indicated the possibility that EGCG could inhibit tumor promotion and be a non-toxic cancer preventive agent.

In 1987, we were first to report that topical applications of EGCG inhibited tumor promotion of teleocidin in a two-stage carcinogenesis experiment on mouse skin initiated with 7,12-dimethylbenz[*a*]anthracene (DMBA) [5]. EGCG also inhibited tumor promotion of another tumor promoter, okadaic acid on mouse skin initiated with DMBA. Okadaic acid is a potent inhibitor of protein phosphatases 1 and 2A (PP1 & PP2A), and it induces tumor promotion through a different mechanism of action from TPA. It was exciting to find that EGCG inhibited tumor promotion through two different mechanisms: Activation of PKC and inhibition of PP1 and PP2A [10]. These results led us to believe that EGCG interrupts the interaction of tumor promoters with their receptors, resulting in prevention of tumor promotion on mouse skin.

Numerous investigators in the world, including us, have attracted attention to the cancer preventive activity of non-toxic EGCG, one constituent of Japanese green tea beverage, and they reported that oral administration of EGCG or green tea extract prevented carcinogenesis in rodents in a wide-range of target organs: the digestive tracts (esophagus, stomach, duodenum and colon), lung, liver, pancreas, kidney, breast, cervix, prostate and skin [6,11,12,13].

Based on our evidence that EGCG, EGC, and ECG have cancer preventive activity, while (−)-epicatechin (EC) is usually inactive, but we found that the combination of EC and each of the cancer preventive catechins enhanced cancer preventive activities, such as induction of apoptosis, and inhibition of cell growth and tumor necrosis factor-α (TNF-α) release from the cells [14,15]. At that time, we demonstrated that TNF-α acts as an endogenous tumor promoter [16]. The results indicate that green tea is most suitable mixture of green tea catechins for cancer prevention, suggesting that green tea beverage is a cancer preventive, more closely tied with our lives than EGCG by itself.

Many Japanese drink green tea every day, and preventive activity of green tea in humans was first shown by a prospective cohort study of the Nakachi’s group at the Saitama Cancer Center Research Institute in Japan. People in Saitama Prefecture, a tea-producing area, drink green tea throughout the day. Nakachi’s group found that individuals who drink over 10 cups of green tea per day showed a 7.3 years delay of cancer onset in females, which is a significant biomarker of cancer prevention [17]. The exciting results of this cohort study led us to find that the cancer preventive amounts of green tea per day are 10 Japanese size cups (120 mL/cup) equivalent to 2.5 g green tea extract. In addition, tablets of green tea extract were prepared by Saitama Prefectural Tea Research Institute, and the preclinical safety trials with 10 cups of green tea supplemented with green tea extract tablets (G.T.E) did not show any serious adverse effects in the participants [6], so that we could move on to double-blind randomized clinical phase II prevention trials: Our cancer prevention strategy consisted of daily green tea beverage supplemented with green tea extract tablets (G.T.E). Drinking 10 cups of green tea supplemented with G.T.E for one year reduced recurrence of colorectal adenomas by 51.6% in patients after polypectomy, in a study conducted at Gifu University by Moriwaki’s group (in collaboration with us [2]. Shin at Seoul National University found similar results using green tea tablets made in Korea [3]. In these clinical trials, no adverse effects were reported. Since then, numerous review articles on the cancer preventive activity of green tea and EGCG have followed [11,13,18,19].

Considering our evidence that green tea significantly prevented recurrence of colorectal adenomas in Japanese patients, we raised two questions: Might it be advantageous for Japanese cancer patients to take green tea catechins and anticancer drugs together? Would the combination enhance efficacy and reduce adverse effects of the drugs? Our experiments provided exciting results showing that the combinations of EGCG and sulindac, and EGCG and tamoxifen synergistically induced apoptosis in human lung cancer cells PC-9 in vitro [14,20]. Since then, numerous investigators have investigated the anticancer activity of the combinations of EGCG or other green tea catechins with anticancer compounds in human cancer cell lines of various cancer types: The anticancer activity of the combination was demonstrated by inhibition of tumor volume in xenograft mouse models implanted using various human cancer cell lines [21,22], and the average reduction in tumor volume in 13 in vivo experiments was 70.3% [21]. All the animal experiments showed that treatment with combinations of EGCG and anticancer compounds was not toxic to rodents during the experiments, and that the combination increased the sensitivity of the anticancer drug to the resistant cancer cells. It is also possible to increase the dose of EGCG more than that used in the experiment. The optimal doses of EGCG and anticancer drugs can be found through the experiments, this is, increasing the dose of EGCG and reducing the dose of anticancer drugs. Since it is now accepted that green tea has cancer preventive activity for humans, the combination of green tea catechins and numerous anticancer compounds will pay greater attention to clinical application of green tea catechins. Now cancer treatment with a combination of green tea catechins and anticancer compounds is becoming an innovative strategy in humans, resulting in improved quality of life without the side effects of anticancer drugs.

## 3. Inhibition of Metastasis with EGCG and Green Tea Catechins

In relation to clinical cancer treatment with green tea catechin, Taniguchi et al. at Kyushu University reported in 1992 that treatment of mice with EGCG in drinking water inhibited lung metastasis in two experimental models: spontaneous (lymphogenous) metastasis from a foot pad by inoculation of B16-BL6 melanoma cells into male C57BL/6 mice, and hematogenous metastasis from the tail vein by injection of B16-F10 melanoma cells in male C57BL/6 mice (Table 1) [23]. The result with the first experiment is clinically important, since lymphogenous metastasis in mice was significantly inhibited by the mice drinking EGCG. Oral administration of EGCG decreased the average numbers of lung nodules, which are accumulated metastasized cancer cells, from 25 to 7 with 0.05% EGCG and from 25 to 10 with 0.1% EGCG (Table 1). The second experiment was conducted as follows: EGCG in drinking water at concentrations of 0.05% and 0.1% was given from 1 day before intravenous (i.v.) injection of B16-F10 cells in male C57BL/6 mice. Three weeks later, the average numbers of lung nodules were >150 for control group, 107 for 0.05% EGCG group and 76 for 0.1% EGCG group, showing that EGCG dose-dependently inhibited hematogenous metastasis of B16-F10 cells into the lung (Table 1).

Another research group reported similar inhibition of lung metastasis of B16-F3m melanoma cells in C56BL/6 mice treated with EGCG, and evidence showed that EGCG inhibited tyrosine phosphorylation of focal adhesion kinase and matrix metallloproteinase-9 [24]. Other reports followed: EGCG inhibited metastasis of human colorectal cancer into the liver in severe combined immunodeficiency (SCID) mice; EGCG-rich green tea polyphenols (GTP) in drinking water inhibited lung metastasis of mouse mammary carcinoma 4T1 cells in BALB/c mice, associated with reduction of tumor growth [25]. Since inhibition of metastasis is one of the most vital subjects in cancer research, this article deals with the inhibitory effects of EGCG and green tea extract on metastasis.

The inhibition of metastasis was further confirmed by unique experimental models as follows: Transgenic mice exhibiting adenocarcinoma of prostate (TRAMP mice) spontaneously developed prostate cancer with high incidence of metastasis, induced by expression of SV40 T antigen, but administration of green tea catechin mixture reduced incidence of prostate cancer from 100% to 13%, and also abrogated metastasis into the lymph nodes, liver and kidney [26]. Another strain of mice, senescence-accelerated mice prone 10 (SAMP10), showed various senescence symptoms, and immune surveillance potential in eight-month-old mice was lower than that in two-month-old mice. Mouse melanoma cells K1735M2 were intravenously injected into eight-month-old SAMP10 mice [27], and the number of metastatic colonies in the lungs of mice given green tea catechins was far less than that in mice given tap water.

Since primary breast cancer can cause secondary tumors in the other side of breast, it is not easy to differentiate metastasis and recurrence of breast cancer in humans. Thus, recurrence of cancer is an important subject to be studied with EGCG and green tea. Decreased recurrence of human breast cancer with increased consumption of green tea was shown by Nakachi and his associates, based on 417 cancer patients at Saitama Cancer Center hospital [28]. For example, in stages I and II breast cancer patients, the group consuming over five cups of green tea per day (average eight cups) showed a lower recurrence rate, 16.7%, and longer disease-free period, 3.6 years, than those consuming fewer than four cups of green tea per day (average three cups), 24.3%, and 2.8 years. However, in stage III breast cancer patients, green tea did not show any decreased recurrence because stage III breast cancer contains more accumulated genetic changes in the cells than are found in stages I and II. EGCG can inhibit tumor promotion on mouse skin by the growth of cells containing activated c-H-*ras* only, but not progression in the advanced stage, which contains numerous genetic changes. Clinically, for stage 1/II breast cancer patients, increased consumption of green tea was closely associated with a smaller number of metastasized axillary lymph nodes, and with increased expression of progesterone and estrogen receptors. The results indicated that green tea prevents the early stage of recurrence even after the removal of the primary cancer. Since the main cause of cancer death is metastasis in humans, we must understand more fully the beneficial effects of EGCG and green tea catechins for prevention of metastasis and recurrence with melanoma, mammary, prostate and colon cancers.

## 4. Sealing Effects of EGCG

Although numerous biochemical and biological studies on EGCG and green tea have revealed multifunctional effects in vitro and in vivo, it is important to determine how a simple compound like EGCG or a mixture of green tea catechins can induce numerous beneficial effects on cancer in humans, such as prevention of cancer, synergistic anticancer effect, and inhibition of metastasis and recurrence. The mechanism of green tea catechins seems to be more complex for cancer cells than the mechanisms of anticancer drugs. Table 2 summarizes the multifunctional effects of green tea catechins: (1) inhibition of receptor binding, cancer cell growth, invasion and migration, angiogenesis, inflammatory cytokines production, proteasomal activity, various enzyme activities, signaling pathways, epithelial-mesenchymal transition (EMT) and spheroid formation of cancer stem cells; (2) induction of apoptosis, cell cycle arrest and phase II enzyme; (3) modification of epigenetic regulation by affecting DNA methyltransferase (DNMT) and histone deacetylase (HDAC), and miRNA expression [10,11,13,18,29,30,31,32,33,34,35,36,37,38,39,40,41]. To understand the diverse effects of EGCG on cancer cells, we introduce here the inhibitory mechanism of tumor promotion on mouse skin.

As previously noted, topical applications of EGCG inhibited two different mechanisms of tumor promotion: Activation of PKC by TPA-type tumor promoters, TPA and teleocidin, and inhibition of PP1 and PP2A by okadaic acid. The studies on specific bindings of ^3^H-TPA and ^3^H-okadaic acid showed that TPA binds to the phorbol ester receptor, while okadaic acid binds to PP1 and PP2A in a particulate fraction of mouse skin [10]. The results showed that a particulate fraction of mouse skin contains the specific receptors of the two tumor promoters. After EGCG was topically applied to mouse skin once, a particulate fraction was prepared from EGCG-treated mouse skin. We found that: The specific bindings of both ^3^H-TPA and ^3^H-okadaic acid had immediately decreased, reaching a minimum level (about 55%) within 10 min, and gradually returning to normal level after 4 h, indicating that EGCG commonly inhibited the interaction of tumor promoters with their receptors. We named this covering of cell membrane by EGCG the “sealing effects of EGCG [10]”. Later, the sealing effects of EGCG were observed in inhibition of the interaction of estrogen and its receptor in breast cancer cells, and also in inhibition of tyrosine phosphorylation of EGF receptor with EGF in A431, and HT29 cells [42].

Shunsaku Kimura at Kyoto University studied the sealing effects of EGCG using a biophysical approach, and demonstrated that EGCG inhibited PKC activation by TPA in the presence of dimyristoylphosphatidylcholine (DMPC) liposome, so probably the interaction of EGCG with phospholipid bilayer membrane affected PKC activation. These results suggest that EGCG causes sealing effects for PKC by inhibiting interaction of various ligands with proteins [43]. Sealing effects of EGCG are further supported by two facts, i.e., the structural and functional similarity between EGCG and chaperones, which interact with various proteins, and the flexible conformations of EGCG [44]. Stochastic conformational analysis in silico found numerous conformations in the catechin molecules: Numbers of flexible conformations are 59 for EGCG, 52 for ECG and 10 for EGC (Figure 1). The flexible conformations of EGCG are strongly associated with “sealing effects”, resulting in numerous biological activities, including inhibition of cell growth and induction of apoptosis in variable cancer types.

## 5. Significance of Biophysical Phenotypes in Cancer Progression and Metastasis

Recent studies using physical biomechanical tools, such as AFM, a microfluidic optical stretcher, and a magnetic tweezer system have revealed that cancer cells are more compliant (smaller elasticity) and more deformable than normal cells of the same tissue [8,45]. In 2007, Cross et al. at UCLA reported that metastatic cells in pleural fluids obtained from patients of lung, breast, and pancreatic cancers have significantly lower average values of Young’s modulus with less stiffness (equivalent to smaller elasticity) than normal mesothelial cells in the body fluids [46]. Similarly, various researchers studied the ratios of Young’s moduli of cancer cells to that of normal cells measured by AFM, and the deformability of cancer cells compared with normal cells as determined by a microfluidic optical stretcher in various cancer cells. Cancer cells taken from the breast, cervix, ovary, bladder, pancreas, stomach, lung and oral cavity showed a lower ratio of Young’s moduli (cancer/normal) about 0.03–0.92 times with less stiffness, and 2–3.5 greater deformability than that of normal cells (Table 3) [46,47,48,49,50,51,52,53,54]. The data showed that human cancer cells of various types have low stiffness as a common biophysical phenotype.

Furthermore, stiffness is a useful biomarker for differentiating between cancer cells and normal cells, and is a distinct metastatic phenotype of cancer cells. For example, mouse B16 melanoma subclones, B16-F10, B16-BL6, and B16-F1, have varied metastatic (cell migration) potentials, but they have similarly rapid growth rates and cancer morphology. Injection of three subclones into tail vein of C57BL/6 mice, B16-F10 cells produced the highest number of metastatic nodules in the lungs, since B16-F10 cells are highly metastatic in mice among these three subclones. B16-BL6 cells produced intermediate numbers of nodules, and B16-F1 cells showed the smallest.

Young’s moduli provided average values of 350.8 ± 4.8 Pa for B16-F10 cells, 661.9 ± 16.5 for B16-BL6 cells and 727.2 ± 13.0 Pa for B16-F1 cells, and the highest metastatic B16-F10 cells showed 0.48 times lower average value of Young’s moduli, indicating that highly metastatic B16-F10 cells have the lowest stiffness (the smallest elasticity), and the lowest metastatic B16-F1 cells have the highest average value of Young’s moduli have the highest stiffness (the largest elasticity) (Table 4) [55]. Thus, stiffness based on Young’s moduli was a good indicator of metastatic potential. Similar experiments were conducted with the other metastatic cancer cells (Table 4): Human melanoma cells WM226-4 were derived from metastatic tissue showed 0.72 ratio of Young’s moduli compared with WM115 derived from primary tumors [56]. Ratio of Young’s moduli for invasive ovarian cancer cells HEY A8 vs. less invasive parental cells (HEY) was 0.56, and primary tongue squamous cell carcinoma (TSCC) cells from patients with metastasis vs. primary cancer cells from patients without metastasis was 0.53 [51,57]. Furthermore, using a magnetic tweezer system, ovarian cancer cells HEY were 10 times more deformable than the least invasive cells, IGROV [58].

All high metastatic cancer cells significantly showed a low average value of Young’s modulus and low stiffness, which correlate well with migration and invasion potential (Table 4). Primary TSCC cells showed that low stiffness is closely associated with expression level of EMT related proteins: High expression level of vimentin and low expression level of E-cadherin observed in TSCC cells are associated with low average value of Young’s moduli and high metastatic potential [57]. Treatment of human gastric cancer cells MKN-1 with TNF-α inducing protein (Tipα), which is a carcinogenic factor of *Helicobacter pylori* and an EMT inducer, reduced average values of Young’s modulus, and increased cell migration (motility) and expression of vimentin, indicating malignant phenotypes [59]. Transforming growth factor-β (TGF-β) is a well-known EMT inducer, and treatment of normal murine mammary gland (NMuMG) cells with TGF-β similarly showed a shift toward lower stiffness (about 3-fold weaker) than with untreated cells [60]. We think low stiffness of cancer cells is a biophysical phenotype of EMT in cancer progression.

It is now well accepted that cancer stem cells or tumor initiating cells drive tumorigenesis, metastasis and cancer progression. Sun et al. at Chongqing University reported that membrane stiffness of cancer stem cells is more soft than that of parental cells in the experiments with enriched liver cancer-stem like cells, named sphere-forming cells (SFCs), derived from human hepatoma cell line MHCC97H. SFCs showed stem cell phenotypes, such as chemoresistance against 5-fluorouracil and cisplatin, and high expression of CD133 and Oct3/4, compared with parental MHCC97H cells [61]. The average values of Young’s moduli were 0.7305 ± 0.196 kPa for MHCC97H, and 0.5824 ± 0.0996 kPa for SFCs. It is important to note that cancer stem cells have 0.8 times softer stiffness than parental cancer cells (Table 4).

**Figure 2:**
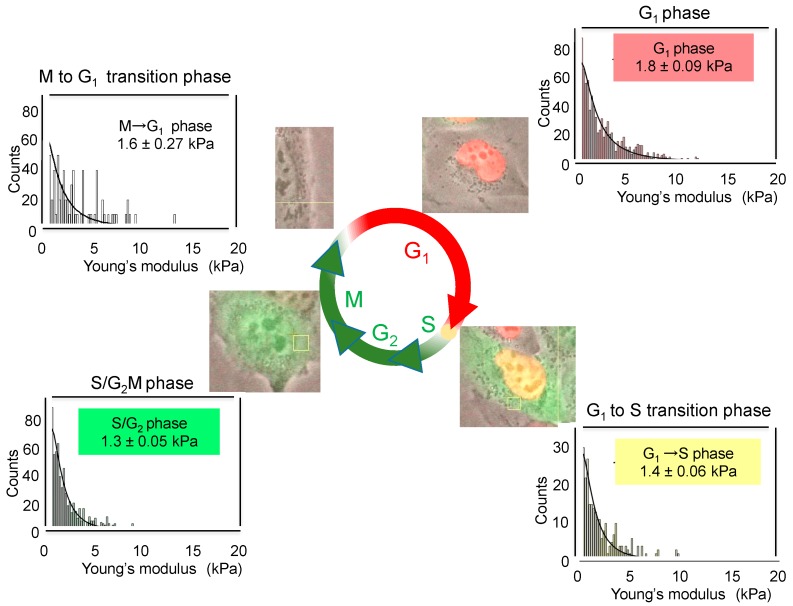
Changes in cell stiffness depending on cell-cycle progression in H1299/Fucci cells. The fluorescent color from red (G_1_ phase), to yellow (G_1_ to S transition phase), to green (SG_2_/M phase) and to no color (M to G_1_ transition phase). Average values of Young’s moduli of cells were determined in each phase by AFM.

The cell cycle induces the changes in membrane stiffness of cells. When the cell cycle progression of live cells was monitored with human lung cancer cell line H1299 expressing Fluorescent ubiquitination-based cell cycle/indicator (H1299/Fucci), the red fluorescent protein expressed by pFucciG_1_-orange, accumulated in G_1_ phase, and the green fluorescent protein expressed by pFucciSG_2_/M-green, accumulated in SG_2_/M phase [62]. Depending on cell cycle progression, H1299/Fucci cells changed the fluorescent color from red (G_1_ phase) to yellow (G_1_ to S transition phase), to green (S/G_2_M phase), and to no color (M to G_1_ transition phase) (Figure 2). According to these changed colors, we determined the average values of Young’s moduli and stiffness of cells in each phase by AFM. Average values changed depending on cell cycle as follows: 1.8 ± 0.09 kPa for G_1_ phase, 1.4 ± 0.06 kPa for G_1_ to S transition phase, 1.3 ± 0.05 kPa for S/G_2_M phase and 1.6 ± 0.27 kPa for M to G_1_ transition phase (Figure 2). Although the differences in the average values are rather small during cell cycle progression, G_1_ phase cells showed a high value of Young’s moduli, indicating rigid stiffness. Therefore, we think that the study of cell cycle by AFM revealed, for the first time, that cell stiffness of individual cancer cells indicates malignant phenotypes, such as proliferation, EMT, migration, and stemness, which are now compiled into “heterogeneity of cancer cells”.

## 6. Biophysical Effects with Green Tea Catechins

### 6.1. Increase in Stiffness of Cancer Cells with Green Tea Extract and EGCG

Gimzewski’s group (James K. Gimzewski) at UCLA first found that treatment of metastatic tumor cells with green tea extract (GTE) dramatically increased cell stiffness of cells, from 0.43 kPa to 2.53 kPa, about 6.2-fold, based on the average values of Young’s modulus of nine tumor cells (Table 5) [63]. All cells obtained from pleural effusion of patients with pancreas, lungs, ovary, and breast cancers showed similar results: EGCG increases stiffness of cancer cells, going from soft to rigid. However, it is important to note that green tea extract did not affect the elasticity of normal mesothelial cells in pleural effusions (1.1-fold) (Table 5). This indicates that green tea extract specifically increased stiffness to large elasticity, which is a biophysical phenotype resulting in cancer prevention. Treatment with green tea extract increased cytoskeletal-F-actin mediated by annexin-I expression and led to rigid stiffness of lung cancer cells A549 [63].

**Figure 3:**
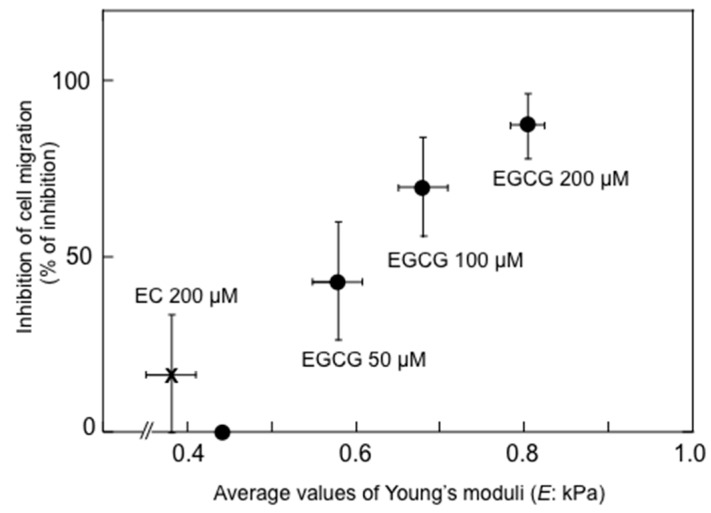
Increase in average values of Young’s moduli and inhibition of cell migration depending on the doses of EGCG in B16-F10 cells. EGCG dose-dependently increased cell stiffness and enhanced inhibition of cell migration, but EC did not affect stiffness or cell migration.

It is important to note that anticancer agents, such as taxol, cisplatin and topotecan increased membrane stiffness of various cancer cells, resulting in changing to large elasticity without any selectivity of cancer cells. We also investigated the relationship between alteration of cell stiffness and inhibition of migration potential of cells treated with EGCG. Treatment of B16-F10 mouse melanoma cells with EGCG dose-dependently resulted in elevated average values of Young’s moduli from 0.44 kPa to 0.58 kPa (50 μM EGCG), 0.68 kPa (100 μM EGCG) to 0.80 kPa (200 μM EGCG), (1.3-fold to 1.8-fold enhancement, associated with large elasticity), and inhibited cell migration, 42.9%, 69.7% and 87.4%, respectively, (Figure 3, Table 5) without any cytotoxic effects [55]. The average value of Young’s moduli of EGCG-treated B16-F10 cells corresponds to that of low metastatic B16-F1 cells (0.72 kPa). An inactive green tea catechin, (−)-epicatechin (EC), at 200 μM did not increase the average value of Young’s moduli indicating little increase in cell stiffness (only 0.8-fold) (Figure 3). Increased cell stiffness in lung cancer cell lines was also observed by treatment with EGCG, from 1.24 to 2.55 kPa for H1299 and from 1.29 to 2.28 for Lu99, 1.8-fold increase (Table 5) [35].

The wound healing assay is a useful method to visualize the inhibitory effects of EGCG on migration of cancer cells. The cells at the leading edge of artificial scratch are actively migrating towards the center of the scratch, and expressing EMT phenotypes, such as Slug and vimentin, compared with those on the other inside parts of cells. EGCG significantly inhibited up-regulation of Slug and vimentin in H1299 and Lu99 cells, suggesting that EGCG blocked EMT induction by stiffening cell membrane (Table 5) [35]. Considering the mechanism of EGCG on inhibition of cell migration, we found that treatment with methyl-β-cyclodextrin (MβCD), which depletes cholesterol from cell membrane and alters membrane organization, significantly inhibited migration (motility) of B16-F10 and H1299 cells, and increased membrane stiffness of cells (about 2–3-fold rigidity) [35,55,64]. The effects were similar to those of EGCG. MβCD inhibits activation of EGF receptor by depletion of cholesterol from membrane, disrupts membrane organization, and causes disruption of lipid rafts [42]. Another group reported that transfection with Ha-Ras^V12^ suppressed expression of caveolin-1 in NIH3T3 cells, decreased membrane stiffness of cells (become softer), and stimulated proliferation and migration of the cells. On the other hand, knockdown of caveolin-1 by shRNA reduced stiffness of NIH3T3 cells [48].

**Figure 4:**
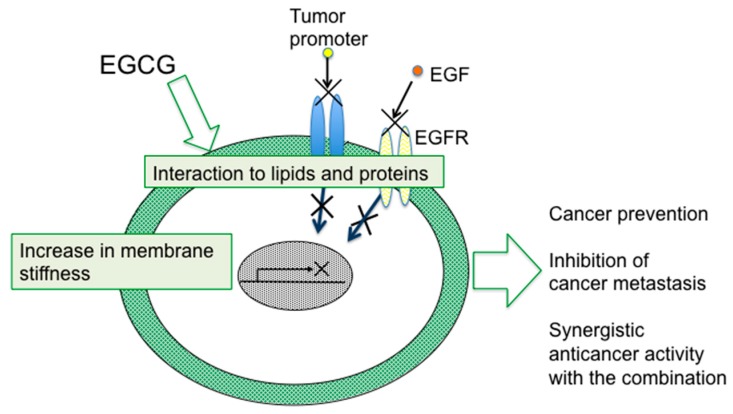
Schematic illustration of biophysical understanding of multifunctions of EGCG in relation to “sealing effects of EGCG”.

Apart from EGCG, Sunyer et al. at the Institute for Bioengineering of Catalonia recently pointed out the significance of a gradient in the stiffness of the extracellular matrix. Their experiments with wound healing showed that clusters of cell placed on the softest region of the gradient showing highest durotaxis exhibited directed migration toward the stiff edge [65]. It is important to determine the active factors making of stiffness gradient, and to study the effects of EGCG on a gradient of stiffness more intensively. All the results indicated that alteration of membrane organization to rigid stiffness with EGCG is a key mechanism for inhibition of cell migration (metastasis) (Figure 4). Thus, “sealing effects of EGCG” comprise the essential interactions of EGCG and lipid membrane.

### 6.2. Other Biophysical Effects of Green Tea Catechins

The study of physical interaction of green tea catechins with the lipid bilayer system representing HepG2 cancer cell membrane indicates that EGCG showed the strongest interaction with lipid bilayer membrane among seven catechins (C, EC, EGC, CG, ECG, EGCG and GCG), because lipid head groups are involved in the formation of hydrogen bonds [66].

Membrane fluidity is the other significant biophysical marker of cell membrane. The presence of hydrophobic and hydrophilic regions in lipid bilayer membrane alters membrane fluidity, which is also influenced by decreased cholesterol and increased unsaturated phosphatidylcholine in membrane. Several groups reported the effects of green tea catechins on membrane fluidity. The effects of EGCG on a model membrane of cancer cells consisting of 20 mol % cholesterol and 80 mol % phosphatidylcholine with 1.0 ratio of the acyl chain 18:1/16:0, and on a model membrane of normal cell consisting of 40 mol % cholesterol and 60 mol % phosphatidylcholine with 0.5 ratio of the acyl chain 18:1/16:0 were studied by fluorescence polarization: EGCG resulted rigid membrane in the model membrane of cancer cells, but had little effect on that of normal cells [67]. So EGCG appears to cause rigidification of fluid lipids. The result correlates well with previously reported in vivo and in vitro results, showing that the effects of EGCG are specifically observed only on cancer cells, not on normal cells [11,63]. The *cis* and *trans* forms of eight green tea catechins showed different reduction levels in membrane fluidity. EGCG in *cis* form showed strong reduction of membrane fluidity [68]. When we look at type 2 diabetes mellitus patients, the peripheral blood mononuclear cells (PBMCs) decreased membrane anisotropy, along with increased insulin resistance parameter. However, treatment of PBMCs with EGCG normalized the membrane anisotropy of the patients, suggesting that beneficial effects of green tea catechins can be anticipated in prevention of diabetic complications, such as cardiovascular disease [69]. These promising results were previously reported by the cohort study in the Saitama Prefecture [17,70].

**Figure 5:**
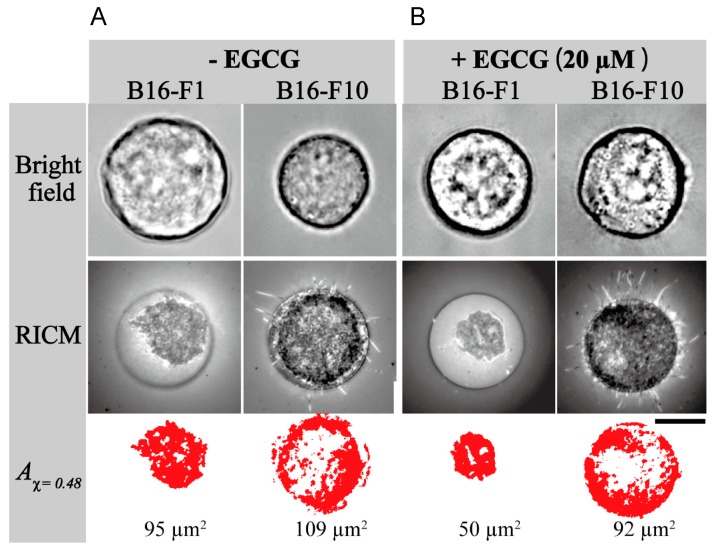

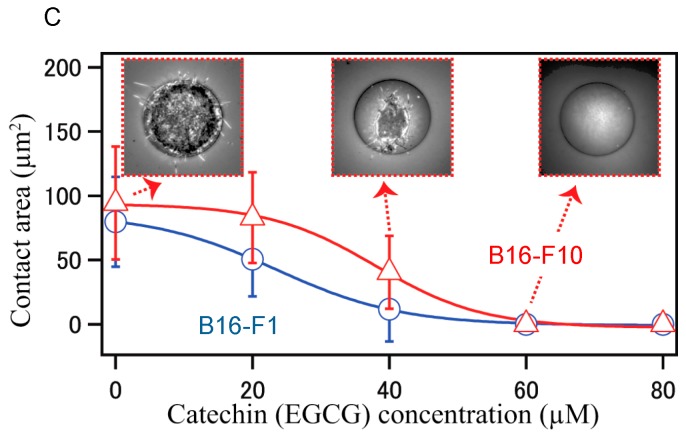
Treatment with EGCG reduced adhesion of low metastatic (B16-F1) and high metastatic (B16-F10) cells. (**A**) Bright field and RICM image show adhesion of B16-F1 and B16-F10 cells on the spot of cell-adhesive SAM substrate. The red area (A_χ_ = 0.48) shows the tight contact area calculated; (**B**) Bright field and RICM image of B16-F1 and B16-F10 cells treated with 20 μM EGCG; (**C**) Dose-dependent reduction of tight contact area in B16-F1 (blue line) and B16-F10 (red line) cells by treatment with EGCG. Reprinted with permission from American Chemical Society [71].

Considering the interaction of green tea catechin with lipid membrane, the interaction alters adhesion of cancer cells, one of the key biophysical phenotypes related to cancer invasion and metastasis. Yoshikawa’s group at Saitama University quantitatively evaluated the adhesion of cancer cells as a tight contact area using both reflection interference contrast microscopy (RICM) and spots of the self-assembled monolayer (SAM)-patterned substrate. RICM visualizes physical contacts between a cell and substrate, and spots of SAM-pattern substrate optimizes the diameter of a cell. These methods made it possible to reliably evaluate the amount of contact area of a single cancer cell on the substrate [71]. The contact area of B16-F1 or B16-F10 cell to the spot of SAM-pattern substrate was clearly visualized in RICM image, but not in the bright field image (Figure 5). The analysis by RICM image revealed that highly metastatic melanoma B16-F10 has slightly larger tight contact area, 109 μm^2^, than low metastatic B16-F1, 95 μm^2^ (A_χ_ = 0.48, Red area in Figure 5A). Treatment with 20 μM EGCG significantly decreased the tight contact area from 95 μm^2^ to 50 μm^2^ for B16-F1 cells, and from 109 μm^2^ to 92 μm^2^ for B16-F10 cells (Figure 5B). Figure 5C clearly shows that EGCG dose-dependently reduced the contact area of both B16-F1 and B16-F10 cells, with slight difference. This suggests that the reduction of adhesion in cancer cells treated with EGCG plays a mechanistic role in inhibition of metastasis. All the results show that EGCG induces a wide range of biophysical effects that are strongly related to the health benefits of green tea, including inhibition of cancer metastasis.

## 7. Conclusions

The biophysical technique AFM made it possible for us to test our proposed mechanism “sealing effects of EGCG”, and to increase our understanding of the beneficial effects of green tea catechins. We discussed here that the inhibitory mechanism of metastasis with EGCG is dependent on the increase in cell stiffness, which is induced by the interaction of cell membrane and proteins. Since green tea catechins have recently attracted attention for preventive effects on cardiovascular disease, diabetic complications, and neurodegenerative diseases including Alzheimer’s and Parkinson’s diseases, numerous clinical trials in addition to cancer prevention, are anticipated all over the world [72,73,74,75,76]. It is important to note that since drinking green tea catechins or green tea extract does not have any serious adverse effects in clinical trials, drinking green tea is a practical method of malady prevention for world people. The recommended amount is 10 cups of green tea per day, equivalent to 2.5 g green tea extract, which is vital for good health. Moreover, our biophysical investigation of EGCG and green tea for cancer prevention and treatment will result in improved quality of life without any adverse effects.

## Figures and Tables

**Table 1:** Inhibition of lung metastasis of B16 melanoma cells by oral administration of EGCG [23].

Groups	Lymphogenous Metastasis with B16-BL6 Cells		Hematogenous Metastasis with B16-F10 Cells	
	Average Number of Lung Nodules	(% of Inhibition)	Average Number of Lung Nodules	(% of Inhibition)
Control	25		>150	
0.05% EGCG	7	72%	107	>29%
0.1% EGCG	10	60%	76	>50%

**Table 2:** Multifunctional effects of green tea catechins.

Effects	Reference
(1) Inhibition of	
Receptor bindings of tumor promoters, hormones, and growth factors (Sealing effects)	[10,31]
Cancer cell growth of numerous cancer cell lines (in vitro and in vivo)	[11,13,18]
Invasion and migration	[13,18,34,35]
Angiogenesis	[18]
Inflammatory cytokines production, such as TNF-α	[11,13,18,31,32]
Proteasomal activity	[18]
Various enzyme activities, such as PKC, ODC, MAP kinases, TERT, and COX	[10,11,13,18]
Signaling pathways of EGFR, HGFR, and FGFR,	[33,42]
Epithelial-mesenchymal transition (EMT)	[34,35]
Spheroid formation of cancer stem cells	[36,37,38]
(2) Induction of	
Apoptosis	[11,13,18,29]
Cell cycle arrest at G_0_/G_1_ or G_2_/M	[11,13,18,30]
Phase II enzymes, such as GS	[18]
(3) Modification of	
Epigenetic regulation by affecting DNMT and HDAC	[18]
miRNA expression, such as miR210, let-7b, miR-1, miR-204	[39,40,41]

**Table 3:** Low stiffness as a common biophysical phenotype in various cancer cells.

Organs	Cancer Cells	Normal Cells	Methods	Ratio of Young‘s Moduli (Cancer/Normal)	Deformability (Cancer/Normal)	Reference
Breast	Metastatic cancer cells from breast cancer patients	Mesothelial cells in pleural fluids	AFM	0.33		[46]
	MCF-7	MCF10	AFM	0.55–0.71		[47]
	MCF-7, MDA-MB 468	M10	AFM	0.18–0.38		[48]
	MCF-7, MDA-MB-231	MCF10	Microfluidic optical stretcher		2.0–3.2	[49]
Cervix	Caski	CRK2614	AFM	0.33		[50]
	SiHa, HeLa	Primary epithelial cells	AFM	0.24–0.41		[48]
Ovary	HEY A8, HEY	IOSE	AFM	0.20–0.36		[51]
Bladder	Hu456, T24, BC3726	Hu609, HCV29	AFM	0.08 0.03–0.14		[52]
	TSGH8301, J82	SVHUC-1	AFM	0.35–0.41		[48]
Pancreas	Metastatic cancer cells from pancreatic cancer patient	Mesothelial cells in pleural fluids	AFM	0.33		[46]
	BxPC-3, PANC-1, ASPC-1, Mia-PaCa-2	HPDE		0.53–0.92		[48]
Stomach	GIST cells from patients	Normal stomach cells	AFM	0.53		[53]
Lung	Metastatic cancer cells from lung cancer patients	Mesothelial cells in pleural fluids	AFM	0.33		[46]
Oral cavity	Oral cancer cells from patients	Epithelial cells from healthy donors	Microfluidic optical stretcher		3.5	[54]

**Table 4:** Low average value of Young’s moduli indicating low stiffness shown in high metastatic cancer cells.

High Metastatic Cancer Cells	Low Metastatic Cancer Cells	Ration of Young’s Moduli/Deformability (High Metastatic /Low Metastatic)	Correlate with	Methods	Reference
Melanoma					
B16-F10	B16-F1	0.48	migration and metastatic potential	AFM	[55]
WM226-4 (derived from metastatic tissue)	WM115 (derived from primary tumor)	0.72		AFM	[56]
Ovary					
HEY A8	HEY	0.56	migration and invasion potential	AFM	[51]
HEY	IGROV	10 times *	migration and invasion potential	Magnetic tweezer system	[58]
Tongue squamous cell carcinoma					
Primary cancer cells from patients with metastasis	Primary cancer cells from patients without metastasis	0.53	migartion and invasion potential high vimentin and low E-cadherin expressions	AFM	[57]
Hepatoma					
Sphere-forming cells derived from MHCC97H	MHCC97H	0.8	migration potential Oct3/4 and CD133 expressions	AFM	[61]

* Deformability.

**Table 5:** Increase of stiffness in cancer cells with green tea extract and EGCG.

Cells	Green Tea Extract or Catechins	Young’s Moduli (kPa) (before → after Treatment)	Fold Increase	Mechanisms	Reference
Tumor cells in pleural effusion from pancreatic (1); lung (3); ovarian (4); and breast (1) cancer patients	Green tea extract	0.43 * → 2.53 * (0.2–0.6) (1.5–3.5)	6.2		[63]
Normal mesothelial cells in pleural effusion	Green tea extract	2.43 ** → 2.60 ** (1.7–2.9) (1.6–3.6)	1.1		[63]
Lung cancer cells A549	Green tea extract	0.23 → 1.0	2.9	Increase of F-actin	[63]
Mouse melanoma cells B16-F10	EGCG	0.44 → 0.80	1.8	Alteration of membrane organization	[55]
	EC	0.44 → 0.36	0.8		[55]
Lung cancer cells H1299	EGCG	1.24 → 2.55	1.8	Alteration of membrane organization Inhibition of EMT	[35]
Lung cancer cells Lu99	EGCG	1.29 → 2.28	1.8	Alteration of membrane organization Inhibition of EMT	[35]

* The average value of Young’s moduli for tumor cells from nine cancer patients; ** The average value of Young’s moduli for mesothelial cells from nine cancer patients.

## Abbreviations

The following abbreviations are uses in the manuscript:

AFM	Atomic force microscopy
DMBA	7,12-dimethylbenz[a]anthracene
DMPC	Dimyristoylphosphatidylcholine
DNMT	DNA methyltransferase
DPPC	Dipalmitoylphosphatidylcholine
EC	(−)-Epicatechin
ECG	(−)-Epicatechin gallate
EGC	(−)-Epigallocatechin
EGCG	(−)-Epigallocatechin gallate
EMT	Epithelial-mesenchymal transition
Fucci	Fluorescent ubiquitination-based cell cycle/indicator
G.T.E	Green tea extract tablets
GTP	Green tea polyphenols
HDAC	Histone deacetylase
i.v.	Intravenous
MβCD	Methyl-β-cyclodextrin
PKC	Protein kinase C
PP1	Protein phosphatase 1
PP2A	Protein phosphatase 2A
RICM	Reflection interference contrast microscopy
SAM	Self-assembled monolayer
SAMP10	Senescence-accelerated mice prone 10
SCID	Severe immunodeficiency
SFCs	Sphere-forming cells
TGF-β	Transforming growth factor-β
Tipα	TNF-α inducing protein
TNF-α	Tumor necrosis factor-α
TPA	12-*O*-Tetradecanoylphorbol-13-acetate
TSCC	Tongue squamous cell carcinoma
